# Detection and Categorization of *Diarrheagenic Escherichia coli* with Auto-microfluidic Thin-film Chip Method

**DOI:** 10.1038/s41598-018-30765-3

**Published:** 2018-08-27

**Authors:** Zhenyu Yun, Lian Zeng, Weijian Huang, Qi Wu, Yiling Fan, Shigang Zheng, Liping Peng, Jiayu Han, Ying Huang, Hang Zhou, Haodong Chen

**Affiliations:** 1 Sichuan Hua Hansan Bio Technology Co., Ltd., #88, Keyuan South Rd., Chengdu, 610041 China; 2 China National Institute of Standardization, #4 Zhichun Rd., Haidian District, Beijing 100088 China; 3 Chengdu CapitalBio Technology Co., Ltd., # 88, Bayi Rd., Chengdu, 610041 China; 4 Shanghai Institute for Food and Drug Control, Shanghai, 201203 China; 5 Chengdu Institute of Biology, Chinese Academy of Sciences, #9, Section 4, Renmin South Rd., Chengdu, 610041 China; 6 National Center of Agriculture Standardization Monitoring and Researching, #1218 Chuangxin 2nd Rd., Songbei science and technology District, Harbin, 150028 China

## Abstract

Diarrheagenic *Escherichia coli* (DEC) causes human diarrhea symptom in both healthy and immunocompromised individuals. An auto-microfluidic thin-film chip (AMTC) instrument integrating one-step multiplex PCR (mPCR) with reverse dot blot hybridization (RDBH) was developed for high-throughput detection of DEC. The novel mPCR method was developed by designing 14 specific primers and corresponding probes. 14 indexes including an endogenous gene (*uidA*) and 13 pathogenic genes (*stx*1, *stx*2, *escV*, *ipaH*, *invE*, *estB*, *lt*, *pic*, *aggR*, *astA*, *bfpB*, *sth* and *stp*) of DEC were detected. This one-step mPCR + RDBH approach is useful for simultaneous detection of numerous target genes in a single sample, whose specificity and availability have been confirmed on the positive control of 11 DEC strains. In addition, with 300 diarrheal stool samples being detected by this method, 21 were found to contain five major DEC strains. Compared with monoplex PCR and previous one-step mPCR approach, this method could detect *ipaH* and *estB*, and compared with current commercial kit, the relevance ratio of DEC detected by the AMTC method was increased by 1% in stool samples. Furthermore, the novel integration AMTC device could be a valuable detection tool for categorization of *E*. *coli*.

## Introduction

Most *E*. *coli* strains are normal inhabitants of the human intestinal tract. Some strains have acquired virulence genes that confer pathogenicity and are clarified as DEC^1^. The major virulence factors are essential for the study of the epidemiology and pathogenicity of DEC infection, such as severe diarrhea, food poisoning and similar outbreaks worldwide. Infections with these kinds of pathogens have therefore aroused increasing concern in the clinical diagnosis of diarrheal disease in recent years. These virulent organisms can be classified into five major categories on the basis of the nature of their infections and pathogenic mechanisms^2–4^, namely, enterohemorrhagic *E*. *coli* (EHEC), enteropathogenic *E*. *coli* (EPEC), enteroinvasive *E*. *coli* (EIEC), enteroaggregative *E*. *coli* (EAEC), and enterotoxigenic *E*. *coli* (ETEC). However, current rates of infections by these important enteric pathogens are probably underestimated to a great extent, because the existing clinical diagnostic methods are unable to distinguish them from normal nonpathogenic flora. To achieve the goal of epidemic prevention and control of DEC, a more reliable procedure is required to identify and categorize DEC isolates.

Many clinical laboratories routinely perform only serotyping assays aiming at detection of DEC. By using primers of virulence genes, the current monoplex PCR or multiplex PCR assay offers the possibility of rapid diagnosis of DEC strains^5,6^. Nevertheless, screening of bacterial isolates for DEC strains requires a large number of individual PCR assays when single primer sets are used^7^. Qualitative detection of DEC is performed by the agarose gelelectrophoresis based on various distributions of different sizes of amplified fragments. So, the number of fragments that can be detected is limited, and the bands of small fragments cannot be recognized very well. Besides, some genes recently reported in the study of the epidemiology and pathogenicity of DEC infection, such as *ipaH* and *estB*^8,9^, were not included in the detection by these methods. Accordingly, it is particularly important and urgent to improve the DEC detection methods.

Recently, one-step mPCR finds wide application in categorization and detection of some bacterial strains^10,11^, and it has been used in some commercial kits as well. This newly-developed method avoids the abundant repeats of individual PCR assays, so it is unnecessary to consider the size of the fragments of PCR products. Moreover, one-step mPCR and RDBH can be combined together with AMTC device^12^. The AMTC method shows some advantages on detection of DEC strains, such as increased test capacity, added indexes like *ipaH* and *estB*, quickened detection, and improved accuracy of detection.

**Figure 1:**
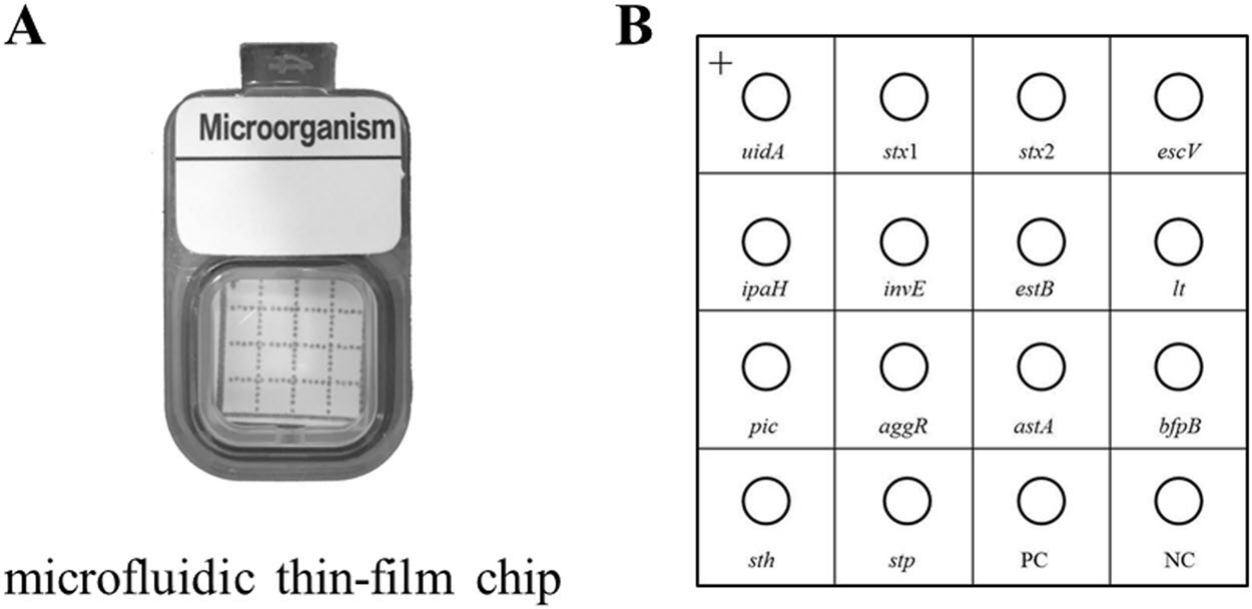
Design of microfluidic thin-film chip. (**A**) Overview of microfluidic thin-film chip. (**B**) Arrangement of DEC probes on the thin-film-based array. *uidA*, β-glucuronidase gene; *stx* (*stx*1, *stx*2), shiga-like toxin I (*stx*1), shiga-like toxin II (*stx*2); *escV*, gene encoding LEE (locus of enterocyte effacement)-encoded type III secretion system factor; *ipaH*, invasive plasmid antigen H-gene; *invE*, invasive plasmid regulator; *estB*, heat-stable enterotoxin b; *lt*, heat-labile enterotoxin; *bfpB*, bundle-forming pilus B; *pic*, protein involved in intestinal colonization; *aggR*, aggregative adhesive fimbriae regulator; *astA*, enteroaggregative heat-stable enterotoxin A; *sth*, heat-stable enterotoxins initially discovered in the isolates from human; *stp*, heat-stable enterotoxins initially discovered in the isolates from pigs; PC, positive control; and NC, negative control.

In this study, 14 specific primers were designed to form one-step multiplex PCR of a newly developed kit, and 14 specific probes were designed accordingly. As shown in Fig. 1A, a square nylon thin-film was divided into 16 small areas using lines. The probes were immobilized on different areas of the square nylon thin-film in line with the surface design in Fig. 1B. Together with the AMTC device, qualitative detection of DEC was successfully established. Further, DEC strains in 300 diarrheal stool samples were detected using the AMTC method.

## Results

### Detection of DEC Strains by Monoplex PCR and Electrophoresis

**Figure 2:**
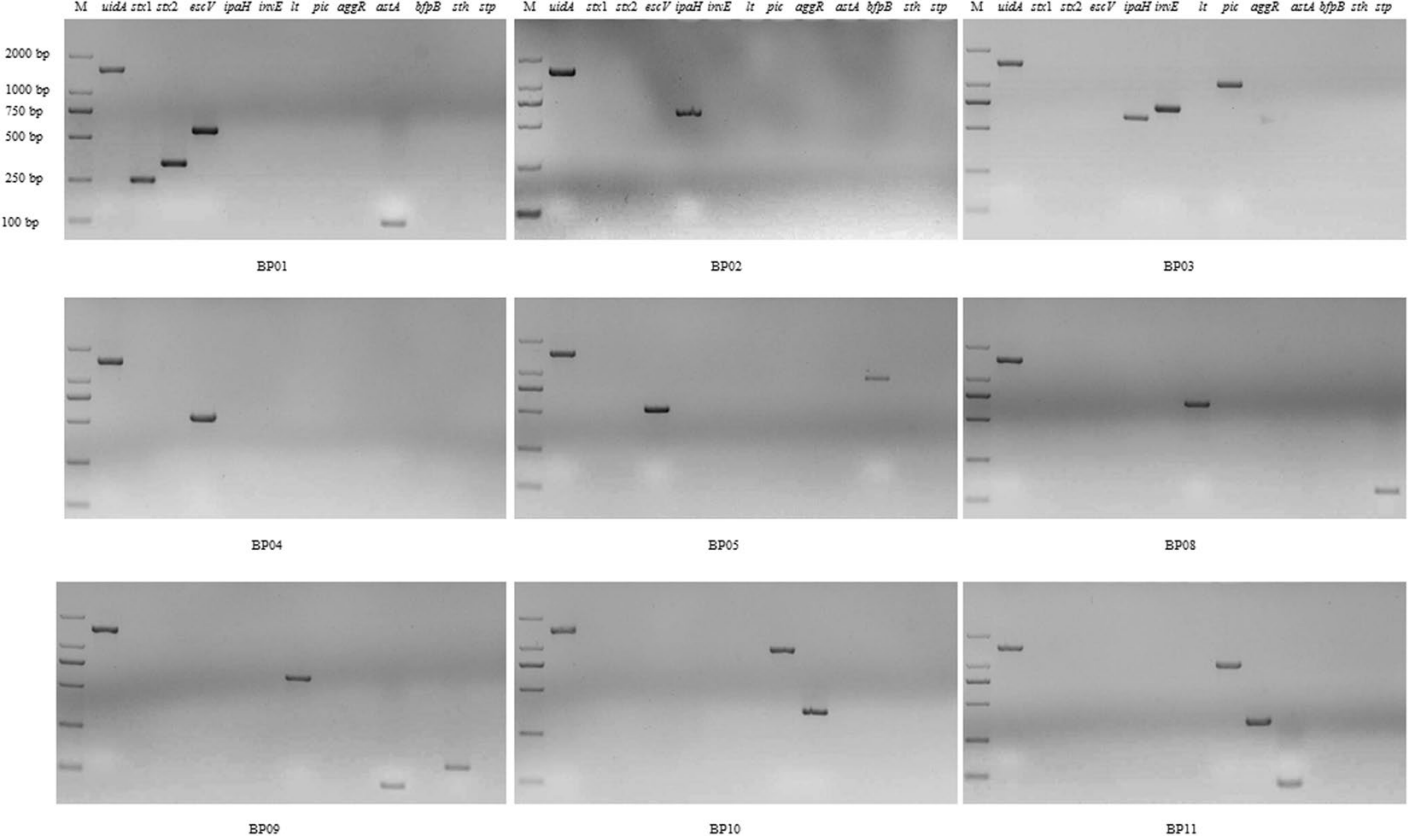
Electrophoretograms of monoplex PCR for DEC strains. BP01 includes *uidA*, *stx*1, *stx*2, *escV* and *astA*, being identified as EHEC; BP02 includes *uidA* and *ipaH*, identified as EIEC; BP03 includes *uidA*, *ipaH*, *invE* and *pic*, identified as EIEC; BP04 includes *uidA* and *escV*, identified as atypical EPEC; BP05 includes *uidA*, *escV* and *bfpB*, identified as typical EPEC; BP06 and BP07 include *uidA*, but no virulence genes; BP08 includes *uidA*, *lt* and *stp*, identified as ETEC; BP09 includes *uidA*, *lt*, *astA* and *sth*, identified as ETEC; BP10 includes *uidA*, *pic* and *aggR*, identified as EAEC; and BP11 includes *uidA*, *pic*, *aggR* and *astA*, identified as EAEC.

By using the specific primers listed in China National Standard (GB 4789.6-2016), the collected DEC strains were identified by monoplex PCR. As shown in Fig. 2, 9 strains were identified positively in 11 DEC strains. BP01 was identified as EHEC; BP02 and BP03 were identified as EIEC; BP04 was identified as atypical EPEC; BP05 was identified as typical EPEC; BP08 and BP09 were identified as ETEC; BP10 and BP11 were identified as EAEC; and BP06 and BP07 were identified as *E*. *coli* (Supplementary Fig. S1).

### Detection of DEC Strains by Previous One-step mPCR and Electrophoresis

**Figure 3:**
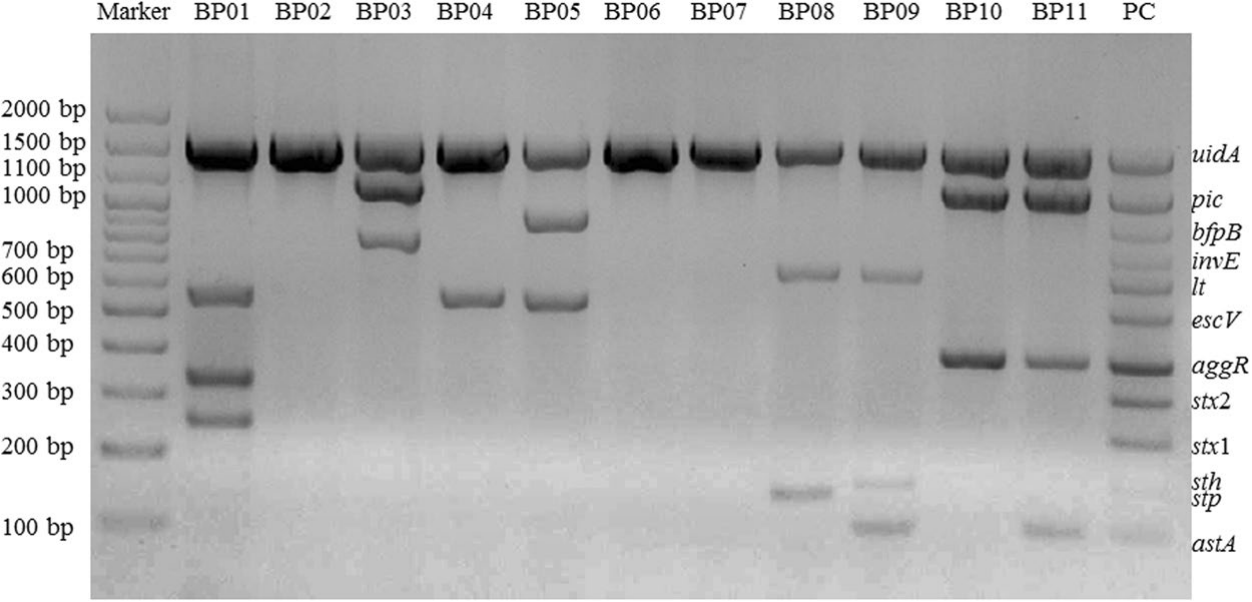
Electrophoretogram of one-step mPCR for DEC strains. BP01 includes *uidA*, *stx*1, *stx*2, *escV* and *astA*, being identified as EHEC; BP02 includes *uidA*, but no virulence genes; BP03 includes *uidA*, *invE* and *pic*, identified as EIEC; BP04 includes *uidA and escV*, identified as atypical EPEC; BP05 includes *uidA*, *escV* and *bfpB*, identified as typical EPEC; BP06 and BP07 include *uidA*, but no virulence genes; BP08 includes *uidA*, *lt* and *stp*, identified as ETEC; BP09 includes *uidA*, *lt*, *astA* and *sth*, identified as ETEC; BP10 includes *uidA*, *pic* and *aggR*, identified as EAEC; and BP11 includes *uidA*, *pic*, *aggR* and *astA*, identified as EAEC.

The above mentioned eleven DEC strains were also detected by a commercial kit, in which one-step mPCR and electrophoresis were mainly used. As shown in Fig. 3, BP01 was identified as EHEC; BP03 was identified as EIEC; BP04 was identified as atypical EPEC; BP05 was identified as typical EPEC; BP08 and BP09 were identified as ETEC; BP10 and BP11 were identified as EAEC; and BP02, BP06 and BP07 were identified as *E*. *coli*. Some *E*. *coli* strains, positive and negative controls were identified (Supplementary Fig. S2).

### Detection of DEC Strains by the Novel One-step mPCR + RDBH

**Figure 4:**
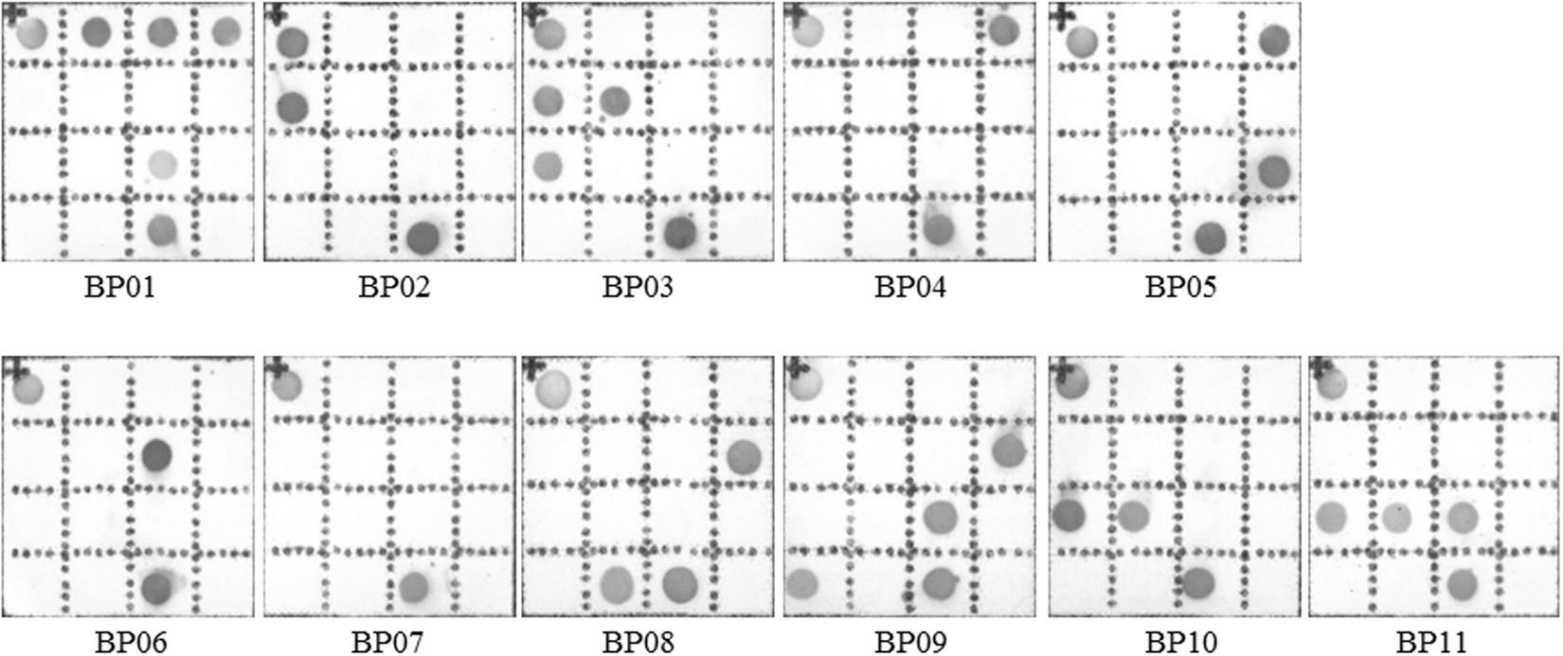
Qualitative detection of DEC strains. BP01 includes *uidA*, *stx*1, *stx*2, *escV* and *astA*, being identified as EHEC; BP02 includes *uidA* and *ipaH*, identified as EIEC; BP03 includes *uidA*, *ipaH*, *invE* and *pic*, identified as EIEC; BP04 includes *uidA* and *escV*, identified as atypical EPEC; BP05 includes *uidA*, *escV* and *bfpB*, identified as typical EPEC; BP06 includes *uidA* and *estB*, identified as ETEC; BP07 includes *uidA*, but no virulence genes; BP08 includes *uidA*, *lt* and *stp*, identified as ETEC; BP09 includes *uidA*, *lt*, *astA* and *sth*, identified as ETEC; BP10 includes *uidA*, *pic* and *aggR*, identified as EAEC; BP11 includes *uidA*, *pic*, *aggR* and *astA*, identified as EAEC.

The detection results of DEC strains by a novel one-step mPCR + RDBH method on AMTC instrument are shown in Fig. 4. BP01 was identified as EHEC; BP02 and BP03 were identified as EIEC; BP04 was identified as atypical EPEC; BP05 was identified as typical EPEC; BP06, BP08 and BP09 were identified as ETEC; and BP10 and BP11 were identified as EAEC; only BP07 was identified as *E*. *coli*.

### Detection of Other Diarrhea Related Strains by the Novel One-step mPCR + RDBH

To test the specificity of the novel one-step mPCR + RDBH method on the AMTC instrument, it was also used to test diarrhea-related strains different from *E*. *coli*. *Salmonella enteric* (CICC 21493), *Vibrio parahaemolyticus* (CICC 21617), *Campylobacter jejuni* (CICC 22936) and *Vibrio cholera* (CICC 23794) could not be detected by any of the AMTC indexes. Only *Shigella flexneri* (CICC 21534) and *Shigella sonnei* (CICC 21535) could be detected at the site of *uidA* and *ipaH* (Supplementary Fig. S3).

### Detection of Diarrheal Stool Samples by Previous One-step mPCR and Electrophoresis

**Figure 5:**
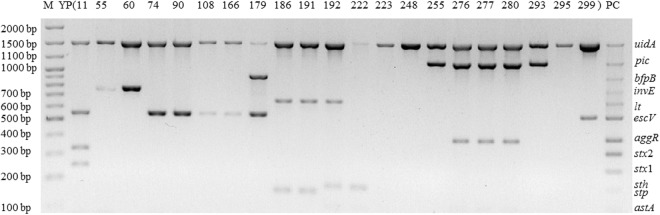
Electrophoretogram of one-step mPCR for diarrheal stool samples. YP11 includes *uidA*, *stx*1, *stx*2 and *escV*, being identified as EHEC; YP55 and YP60 include *uidA* and *invE*, identified as EIEC; YP74, YP90, YP108, YP166 and YP299 include *uidA* and *escV*, identified as atypical EPEC;YP179 includes *uidA*, *escV* and *bfpB*, identified as typical EPEC; YP186 and YP191 include *uidA*, *lt* and *stp*, identified as ETEC; YP192 includes *uidA*, *lt* and *sth*, identified as ETEC; YP222 includes *uidA* and *sth*, identified as ETEC; YP223,YP248 and YP295 include *uidA*, but no virulence genes; YP255 and YP293 include *uidA* and *pic*, identified as EAEC; and YP276, YP277 and YP280 include *uidA*, *pic* and *aggR*, identified as EAEC.

By using the previous one-step mPCR and electrophoresis listed in the current commercial kit, 18 diarrheal stool samples were found to contain DEC strains among a total of 300 samples. The positive rate was 6%. Part of results are shown in Fig. 5, indicating that EHEC was identified positively in one sample; EIEC was identified positively in two samples; typical EPEC was identified positively in one sample; atypical EPEC was identified positively in five samples; ETEC was identified positively in four samples; and EAEC was identified positively in five samples.

### Detection of Diarrheal Stool Samples by the Novel One-step mPCR + RDBH

**Figure 6:**
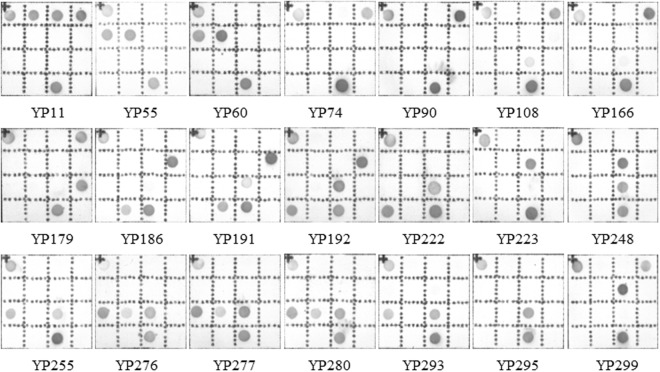
Qualitative detection of DEC strains in diarrheal stool samples by RDBH on AMTC instrument.YP11 includes *uidA*, *stx*1, *stx*2 and *escV*, being identified as EHEC; YP55 and YP60 include *uidA*, *ipaH* and *invE*, identified as EIEC; YP74 and YP90 include *uidA* and *escV*, identified as atypical EPEC; YP108 and YP166 include *uidA*, *astA* and *escV*, identified as atypical EPEC;YP179 includes *uidA*, *escV* and *bfpB*, identified as typical EPEC; YP186 includes *uidA*, *lt* and *stp*, identified as ETEC; YP191 includes *uidA*, *astA*, *lt* and *stp*, identified as ETEC; YP192 includes *uidA*, *lt*, *astA* and *sth*, identified as ETEC;YP222 includes *uidA*, *astA* and *sth*, identified as ETEC;YP223 includes *uidA* and *estB*, identified as ETEC; YP248 includes *uidA*, *astA* and *estB*, identified as ETEC; YP255 and YP293 include *uidA*, *astA* and *pic*, identified as EAEC; YP276, YP277 and YP280 include *uidA*, *pic*, *aggR* and *astA*, identified as EAEC; YP295 includes *uidA* and *astA*, but no virulence genes; and YP299 includes *uidA*, *estB and escV*, identified as atypical EPEC and ETEC.

These 300 diarrheal stool samples were also identified by the novel one-step mPCR + RDBH method on the AMTC instrument, where, 21 samples were detected to contain DEC strains. The positive rate was 7%. As shown in Fig. 6, EHEC was identified positively in one sample; EIEC was identified positively in two samples; typical EPEC was identified positively in one sample; atypical EPEC was identified positively in four samples; ETEC was identified positively in six samples; EAEC was identified positively in six samples; and ETEC+ atypical EPEC were identified simultaneously in one sample. Compared with current commercial kit, the relevance ratio of DEC detected by the AMTC method was increased by 1% in stool samples.

## Discussion

With more detection indexes and improved detection rate of DEC, the AMTC method is able to detect 14 indexes in a single reaction. This would avoid the probability of undetected *ipaH* and *estB*. Compared with monoplex PCR, the AMTC method identified BP06 as ETEC, just owing to *estB*. Compared with commercial kit, the AMTC method identified BP02 as EIEC, and BP06 as ETEC, just owing to *ipaH* and *estB*. For the detection of diarrheal stool samples, compared with commercial kit, the AMTC method identified YP223, and YP248 as ETEC, just owing to *estB*; identified YP295 as EAEC, just owing to *astA*; and identified YP299 as ETEC+ atypical EPEC, just owing to *estB*.

The AMTC method could be used to distinguish sub-type of DEC. Some researchers reported that *astA* encoded the *EAST*1, but *astA* is widely distributed in DEC, and its role in the pathogenicity of this organism remains unclear^13^. Some researchers reported that a strain harboring *astA* was associated with a waterborne outbreak of diarrhea in Japan^14^. So, further studies are needed to evaluate the significance of *EAST*1 as a virulence factor of DEC. This study showed 15 clinical strains contained *astA*, while 7 of them contained 3 *sth*, 1 *stp* and 3 *escV*. Obviously, *astA* is common among EHEC, EPEC, ETEC and EAEC, and each sample should be tested separately with specific primers such as *stx*1, *stx*2, *escV*, *estB*, *lt*, *pic*, *aggR*, *astA*, *bfpB*, *sth* and *stp* by monoplex PCR method. However, these processes could be completed using the newly-developed AMTC method in a single reaction. Some researchers believed that EAEC possesses both *pic* and *aggR* present in the virulence plasmid pAA^15,16^. Some researchers identified a few *pic*-positive and *aggR*-negative strains, and vice versa^17^. Reportedly, some researchers used only *aggR* for detecting pAA^10^. However, we found a positive single virulence gene and coexistence of two positive genes in the detection of diarrhea samples about EAEC.

The AMTC method possesses high efficiency and high throughput. For example, *EscV* is common between EHEC and EPEC, and in order to distinguish these two pathogens, *bfpB* and *stx*1 + *stx*2 must also be run by monoplex PCR method. Meanwhile, when *bfpB* and *stx*1 + *stx*2 are negative, the results can be identified as atypical EPEC, with positive *bfpB* being identified as typical EPEC, and positive *stx*1 + *stx*2 identified as EHEC. The results obtained by us show this method is able to perform both simultaneous amplification of virulence genes from *E*. *coli* isolates and simultaneous differentiation of the 5 categories of DEC.

The AMTC method has the advantages of good repeatability and stability. Two new indexes, *ipaH* and *estB* basing on monoplex PCR, and a multiplex PCR diagnostic kit, have been added to this method. Compared with the previous one-step mPCR, segment length of the PCR products for the novel one-step mPCR was basically the same, thus it could reduce competition between primers. Compared with electrophoretic detection, the fragments of poor amplification could also be detected with such RDBH method, resulting in a stable detection. Furthermore, one-step mPCR integrating with RDBH allows it to improve repeatability and stability of the detection results. Accordingly, the AMTC method would be a valuable contribution to the routine diagnostic laboratory tests while providing important information for the epidemiological and other studies.

## Materials and Methods

### Bacterial Strains

A total of eleven DEC strains used in this study were purchased from China Center of Industrial Culture Collection (CICC). They were *Escherichia coli EHEC O 157:H7* (CICC 21530) containing Shiga-like toxin I (*stx*1), Shiga-like toxin II (*stx*2), *eae* and *escV*^18^, *Escherichia coli EPEC* (CICC 10664) containing *eae* and *escV*, where EPEC can be classified as typical or atypical one based on the production of bundle-forming pili (*bfp*) encoded by the Escherichia adherence factor (EAF) gene^15^, *Escherichia coli EIEC* (CICC 10662) containing *ipaH* and *invE*^19–22^, *Escherichia coli ETEC O78:K80* (CICC 10421) and *O126: K71* (CICC 10415) containing the heat-labile toxin (*LT*) and the heat-stable toxin (*ST*)^22–25^, *Escherichia coli EAEC* purchased from Deutsche Sammlung von Mikroorganismen und Zellkulturen (DSMZ), and *it* (DSMZ 10974) containing *aggR*, *pic* and enteroaggregative heat-stable enterotoxin *EAST*1 (*astA*)^26–28^. Some other diarrhea strains different from *E*. *coli* in this study were *Salmonella enterica subsp*. *Enterica serovar Choleraesuis* (CICC 21493), *Shigella flexneri* (CICC 21534), *Shigella sonnei* (CICC 21535), *Vibrio parahaemolyticus* CICC 21617), *Campylobacter jejuni* (CICC 22936) and *Vibriocholera* (CICC 23794).

### Stool samples

A total of 300 diarrheal stool samples were collected from National Institute for Communicable Disease Control and Prevention, Chinese Center for Disease Control and Prevention. The samples were stored at −80 °C.

### Preparation of DNA Templates

All DNA samples used in this study were extracted by QIAamp DNA Mini Kit (QIAGEN, Germany). The templates used for PCR comprise the DNA of *EHEC O157:H7*, *EPEC*, *EIEC*, *ETEC O78:K80*, *ETEC O126: K71*, *EAEC*, some other DEC strains, some diarrhea strains differing from *E*. *coli*, and diarrheal stool samples. The final concentration of DNA in extracts from bacterial strains and stool samples was 25 ng/µL.

### Probes and Primers used for Detection of DEC

The oligonucleotide probes used in the AMTC corresponding to the reverse primers in one-step mPCR of a newly developed kit are listed in Table 1. An aminolinker C6 was added as a 5′-modified group to provide spaces between probes and immobilization substrates. The oligonucleotide primers used in monoplex PCR were collected from the China National Standard GB 4789.6–2016, being listed in Table 2. The final concentration of each primer used in the PCR amplification system depends on the GB 4789.6–2016. The primers used in a multiplex PCR diagnostic kit (a commercial kit currently used) were the components in the kit. The oligonucleotide primers used in a newly developed kit were designed by Primer premier v5.0, as shown in Table 3. The 5′ ends of the reverse primers were modified by a biotin group. Among them, the proportion of forward and reverse primers for each pair primers was 2:3. The final concentration of each primer in mixed PCR primers should be 10 µM. The predicted length of PCR products was ranged between 100 and 300 bp. All the primers and probes were synthesized by Sangon Biotech (Shanghai) Co., Ltd.

**Table 1:** Probes Used by RDBH on AMTC Instrument.

Gene	Probes Sequence^a^
*uidA*	P: 5′-NH2-CCGGGAATGGTGATTACCGACGAAA
*stx*1	P: 5′-NH2-TGTTACCTTTCCAGGTACA
*stx*2	P: 5′-NH2-CTGAAACTGCTCCTGTGT
*escV*	P: 5′-NH2-TGGCGATTATTCCTGGCTTTCCTAC
*ipaH*	P: 5′-NH2-CGCCTTTCCGATACCGTCTCTGCA
*invE*	P: 5′-NH2-CAGCAAAAGAGCATAGCATCCGAGA
*estB*	P: 5′-NH2-CAACAGTGACAACGGAGGCGA
*lt*	P: 5′-NH2-GATGATACTTGTAATGAGGAGA
*pic*	P: 5′-NH2-TTCTGCAGACGGTGGTTACA
*aggR*	P: 5′-NH2-GGAATATCAAAAGTAGATGC
*astA*	P: 5′-NH2- AACAGCCTGCGCTTCGTGTCATGG
*bfpB*	P: 5′-NH2-TCAGTTCGGACAGCAATAGC
*sth*	P: 5′-NH2-CTAAACCAGCAGGGTCTTCAA
*stp*	P: 5′-NH2-AATCAGAAAATATGAACGACAC
Positive-probe	5′-NH2-GCATCCAGATCAGAAGCAATAATGA
Negative-Probe	5′-NH2-CCCTCGGGTTAATGCGCGATTGTCAC

^a^5′- Modified group: Amino linker C6.

**Table 2:** Primers Used in Monoplex PCR.

Gene	Primer Sequence^a^	Amplicon Size (bp)
*uidA*	F: ATGCCAGTCCAGCGTTTTTGC	1487
	R: AAAGTGTGGGTCAATAATCAGGAAGTG	
*stx*1	F: CGATGTTACGGTTTGTTACTGTGACAGC	244
	R: AATGCCACGCTTCCCAGAATTG	
*stx*2	F: GTTTTGACCATCTTCGTCTGATTATTGAG	324
	R: AGCGTAAGGCTTCTGCTGTGAC	
*escV*	F: ATTCTGGCTCTCTTCTTCTTTATGGCT	544
	R: CGTCCCCTTTTACAAACTTCATCGC	
*ipaH*	F: TTGACCGCCTTTCCGATACC	647
	R: ATCCGCATCACCGCTCAGAC	
*invE*	F: CGATAGATGGCGAGAAATTATATCCCG	766
	R: CGATCAAGAATCCCTAACAGAAGAATCAC	
*lt*	F: GAACAGGAGGTTTCTGCGTTAGGTG	655
	R: CTTTCAATGGCTTTTTTTTGGGAGTC	
*pic*	F: AGCCGTTTCCGCAGAAGCC	1111
	R: AAATGTCAGTGAACCGACGATTGG	
*aggR*	F: ACGCAGAGTTGCCTGATAAAG	400
	R: AATACAGAATCGTCAGCATCAGC	
*astA*	F: TGCCATCAACACAGTATATCCG	102
	R: ACGGCTTTGTAGTCCTTCCAT	
*bfpB*	F: GACACCTCATTGCTGAAGTCG	910
	R: CCAGAACACCTCCGTTATGC	
*sth*	F: TGTCTTTTTCACCTTTCGCTC	171
	R: CGGTACAAGCAGGATTACAACAC	
*stp*	F: CCTCTTTTAGYCAGACARCTGAATCASTTG	157
	R: CAGGCAGGATTACAACAAAGTTCACAG	

^a^Primer sequences were collected from the Chinese National Standard GB4789.6–2016.

**Table 3:** Primers Used in Multiplex PCR.

Gene	Primer Sequence^a^	Amplicon Size (bp)
*uidA*	F: GTCACGCCGTATGTTATTGCC	189
	R: 5′biotin-CGGCGTGGTGTAGAGCATT	
*stx*1	F: AAGAGCGATGTTACGGTTTGT	172
	R: 5′biotin- GTCAGGCAGGACACTACTCAA	
*stx*2	F: GTTCAGTGGTAATACAATGACCAG	204
	R: 5′biotin- TACTCCGGAAGCACATTGCTGA	
*escV*	F: GATGCTTTAGTTGCCCAGAT	220
	R: 5′biotin- CCGCCAGAAACAAGAAGACC	
*ipaH* ^b^	F: TTCCTTGACCGCCTTTCC	131
	R: 5′biotin- TCAGCAGCAACAGCGAAA	
*invE* ^b^	F: GCAGGAGCAGATCTTGAAG	208
	R: 5′biotin- GAAAGGCACGAGTGACTTTC	
*estB*	F: CACAACAGTGACAACGGAGGC	145
	R: 5′biotin- CCGGCAAAGCTATTGGAAAA	
*lt*	F: TCCCACCGGATCACCAAG	124
	R: 5′biotin- GTGCTCAGATTCTGGGTCTCC	
*pic*	F: CAAACGTATGGGTGACCTGC	191
	R: 5′biotin- TGCTGTCGGTATAGGTCATCG	
*aggR*	F: CGCCTAAAGGATGCCCTGAT	107
	R: 5′biotin- ACAGAATCGTCAGCATCAGCTA	
*astA*	F: TGCCATCAACACAGTATATCCG	102
	R: 5′biotin- ACGGCTTTGTAGTCCTTCCAT	
*bfpB*	F: TTCAAACGAGGAAACTAAACGC	158
	R: 5′biotin- AATCGAATTTCAACTCTGCTCC	
*sth*	F: TTCACCTTTCCCTCAGGATG	172
	R: 5′biotin- ATAGCACCCGGTACAAGCAG	
*stp*	F: ACTGAATCACTTGACTCTTCA	144
	R: 5′biotin- AGCACAGGCAGGATTACAAC	
Positive-olig	5′biotin-CTGGTACTTTGGACACTCGTTCTTC	/

^a^Reverse primers were 5′-modified by biotin.

^b^The *ipaH* and *invE* genes were targeted for detection of both *Shigella* and EIEC.

### Monoplex PCR

PCR amplifications were performed as follows: 2 µL of bacterial extract was added to the reaction mixture with a final volume of 20 µL containing 10 µL Premix Taq™ (Takara, Code No. RR902A), 1 µL PCR primer mix and 7 µL ddH_2_O.These mixtures were pre-denatured at 94 °C for 5 min and then amplified for 30 cycles by using a thermal cycler (Model: BIORAD T100). Each cycle was composed of denaturation at 94 °C for 30 s, annealing at 63 °C for 30 s, and extension at 72 °C for 1.5 min. A final extension step was performed at 72 °C for 5 min, and the tubes were rapidly cooled to 4 °C. Qualitative detection of DEC was accomplished by the 2% agarose gel electrophoresis.

### One-step mPCR

One-step mPCR was performed by using a multiplex PCR diagnostic kit and a newly developed kit, respectively. PCR mix of the multiplex PCR diagnostic kit, with a final volume of 25 µL, included 2 × PCR Buffer 12.5 µL, 10 × Multiplex Assay 2.5 µL, 25 × PCR Enzyme 1 µL, 2 µL of bacterial extract, and 7 µL of ddH_2_O. These mixtures were pre-denatured at 95 °C for 4 min and then amplified for 30 cycles by using a thermal cycler (Model: BIORAD T100). Each cycle was composed of denaturation at 95 °C for 30 s, annealing at 62 °C for 30 s, and extension at 72 °C for 90 s. A final extension step was conducted at 72 °C for 5 min, and the tubes were rapidly cooled to 4 °C. Qualitative detection of DEC was accomplished by the 2% agarose gel electrophoresis about a multiplex PCR diagnostic kit. PCR mix of the newly developed kit, with a final volume of 20 µL, included 10 µL of 2 × KAPA2G Fast Multiplex Mix (KAPA Biosystems, USA), 1 µL of mixed PCR primers, 2 µL of bacterial extract, and 7 µL of ddH_2_O. These mixtures were preheated at 37 °C for 5 min, pre-denatured at 95 °C for 3 min and then amplified for 35 cycles by using a thermal cycler (Model: BIORAD T100). Each cycle was composed of denaturation at 95 °C for 15 s, annealing at 55 °C for 30 s, and extension at 68 °C for 15 s. A final extension step was conducted at 68 °C for 3 min, and the tubes were rapidly cooled to 4 °C. Qualitative detection of DEC was accomplished by RDBH about a newly developed kit.

### Microfluidic thin-film chip

DNA probes were spotted and immobilized on a nylon thin-film according to the surface design. This step can be performed manually or by a machine. Then, the nylon thin-film probes spot array was incubated in 80 °C for 1 h, and it was placed in a small cell with two small holes. The microfluidic thin-film chip consisted of three main parts: a square nylon thin-film, a small cell with two small holes, and two microfluidic tubes. The small cell was a small square plastic box with a sealing ring on the inner side of the lid. When a square nylon thin-film was placed into the small box, the lid was closed, forming a closed cell. PCR products were added to the reaction cell through the microfluidic tube. When the PCR products passed over the surface of the square nylon thin-film containing the probe array, the target DNA in the PCR products was captured by the sensing surface DNA base pairing between the probes and target DNA^12^.

### Qualitative Detection of DEC with RDBH

The AMTC instrument consisted of four main parts: a machine shell, micro-reaction cell, sample needle with pump, and sample and waste liquid plate. The eight microfluidic thin-film chip cells were placed on the micro-reaction cell of the AMTC device (*Sichuan Hua Hansan Bio Technology Co*., *Ltd*. *#39*, *Fucheng West Rd*., *Chengdu*, *610041*, *China*). PCR products were added as samples to the sample plate of the AMTC device, and then the single-stranded DNA (Positive-Oligo, 10 μmol/L) complementary to the positive control (PC) probe was added to each PCR product sample for quality control. The hybridization buffer, cleaning buffers, enzyme buffer, and dyeing agent (NBT and BCIP) were placed in sample plate, with the signal being visualized with streptavidin-alkaline phosphatase color development kit (ZSGB-BIO, China). The sample needle with pump operated with an up and down movement and took different samples by rotating the sample plate. Under the control of an automatic hybridization program, the AMTC instrument could add samples and perform the reaction, washing, and coloring steps. The running procedures and time of the AMTC instrument were listed in Table 4.

**Table 4:** The running procedures of the AMTC instrument.

Procedure	Reagent	Temperature (°C)	Time (min)
deactivation	deactivation solution	37	8
deactivation cleaning	deactivation cleaning solution	60	5
hybridization	hybridization solution	45	45
hybridization cleaning	hybridization cleaning solution	52	5
hybridization cleaning	hybridization cleaning solution	52	5
enzyme labelling	enzyme solution	42	30
enzyme cleaning 1	enzyme cleaning solution 1	42	5
enzyme cleaning 2	enzyme cleaning solution 2	37	5
enzyme cleaning 2	enzyme cleaning solution 2	37	5
color rendering	Color-substrate solution	37	10
chromogenic cleaning	distilled water	/	2
chromogenic cleaning	distilled water	/	2
chromogenic cleaning	distilled water	/	2
Interpretation of results	/	/	/

### Statistical analysis

The criterion for classification of diarrhoea *E*. *coli* was shown in Table 5. According to the criterion for classification, positive samples were identified. Compared with the total number of samples, the positive rate of different methods can be calculated.

**Table 5:** The criterion for classification of diarrhoea *E*. *coli*.

Pathogenicity	virulence gene	Positive	
EPEC	*escV*, *astA*, *bfpB*	*uidA* (+/−)	typical EPEC: *escV* (+), *bfpB* (+), *stx*1 (−), *stx*2 (−)
			atypical EPEC: *escV* (+), *bfpB* (−), *stx*1 (−), *stx*2 (−)
EIEC	*ipaH*, *invE*, *pic*	*uidA* (+/−)	*ipaH* (+), *invE* (+/−)
ETEC	*estB*, *lt*, *sth*, *stp*, *astA*	*uidA* (+/−)	One or more than 1 of *estB*, *lt*, *sth* and *stp* (+)
EHEC	*stx*1, *stx*2, *escV*, *astA*	*uidA* (+/−)	one or more than 1 of *stx*1 and *stx*2 (+), bfpB (−)
EAEC	*Pic*, *aggR*, *astA*	*uidA* (+/−)	One or more than 1 of *pic*, *aggR* and *astA* (+)

## Electronic supplementary material

Dataset 1
