# Supplementary material for: Detection and Categorization of Diarrheagenic Escherichia coli with Auto-microfluidic Thin-film Chip Method
Source: Sci Rep. 2018 Aug 27;8:12926. doi: 10.1038/s41598-018-30765-3 (PMC6110803; doi:10.1038/s41598-018-30765-3)
Supplement: Supplementary file 1 — Dataset 1 [file 41598_2018_30765_MOESM1_ESM.doc]

**Supporting Information**

**Detection and Categorization of *Diarrheagenic Escherichia coli* with Auto-microfluidic Thin-film Chip Method**

Zhenyu Yun^2#^, Lian Zeng^1#^, Weijian Huang^3^, Qi Wu^2^, Shigang Zheng^4^, Liping Peng^5^, Jiayu Han^1^, Ying Huang^1^, Hang Zhou^1^, Haodong Chen ^1^*

*1. Sichuan Hua Hansan Bio Technology Co., Ltd. #39, Fucheng West Rd., Chengdu, 610041, China*

*2. China National Institute of Standardization, #4, Zhichun Rd.,* *Haidian District, Beijing 100088, China*

*3. CapitalBio Technology (Chengdu) Co., LtD*

*4. Chengdu Institute of Biology, Chinese Academy of Science, Chengdu, 610041, China*

*5. National Center of Agriculture Standardization Monitoring and Researching, #1218 Chuangxin 2nd Rd., Songbei science and technology District, Harbin 150028, China*

^#^ These authors contributed equally to this work.

*Corresponding author (email: chenhaodong301@163.com)


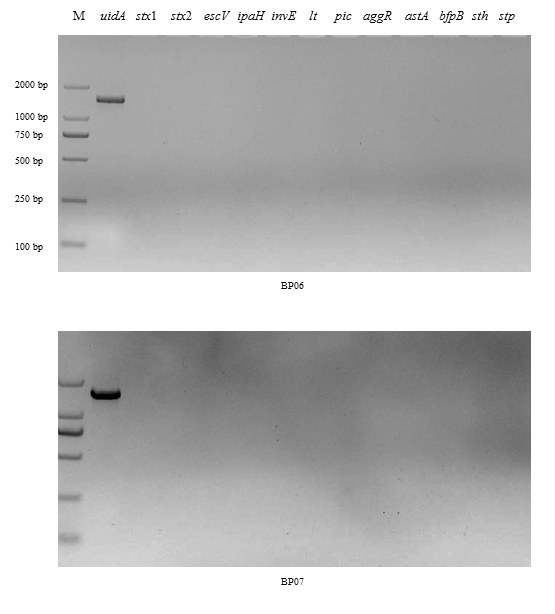


**Figure S1** BP06 and BP07 were identified by monoplex PCR. BP06 and BP07 include *uidA*, but no virulence genes.


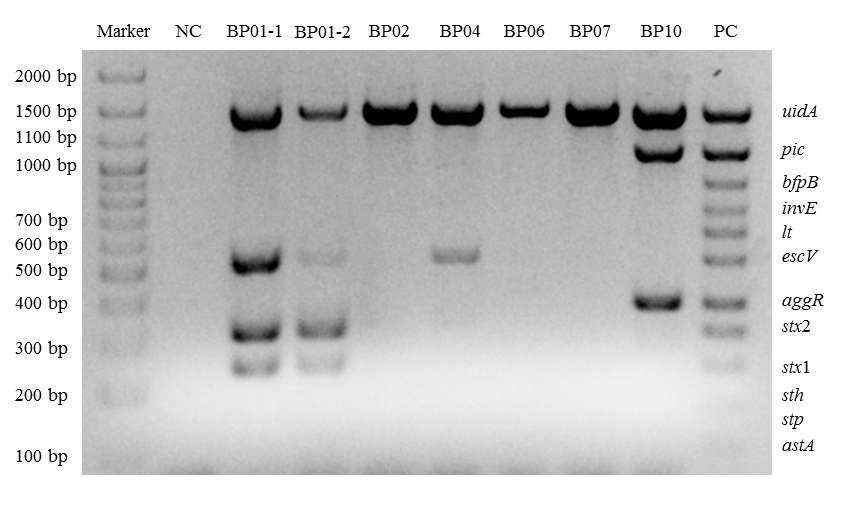


**Figure S2** Electrophoretogram of one-step mPCR for some *E. coli* strains. BP01-1 and BP01-2 includes *uidA*, *stx*1, *stx*2, *escV* and *astA*, being identified as EHEC; BP02 includes *uidA*, but no virulence genes; BP04 includes *uidA and escV*, identified as atypical EPEC; BP06 and BP07 include *uidA*, but no virulence genes; BP10 includes *uidA*, *pic* and *aggR*, identified as EAEC.


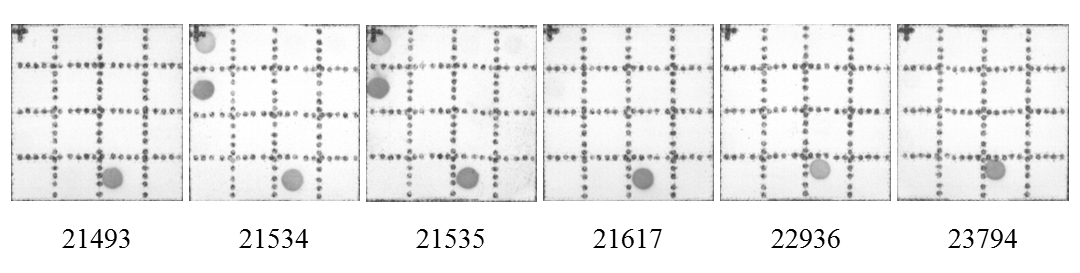


**Figure S3** Qualitative detection of diarrhea-related strains different from *E. coli*.
